# Editorial: Updates in ocular therapeutics and surgery, volume IV

**DOI:** 10.3389/fmed.2025.1685404

**Published:** 2025-09-04

**Authors:** Georgios D. Panos, Horace Massa

**Affiliations:** ^1^First Department of Ophthalmology, AHEPA University Hospital, School of Medicine, Aristotle University of Thessaloniki, Thessaloniki, Greece; ^2^Division of Ophthalmology and Visual Sciences, School of Medicine, University of Nottingham, Nottingham, United Kingdom; ^3^Department of Ophthalmology, Geneva University Hospitals, Geneva, Switzerland

**Keywords:** cornea, ocular therapeutics, ophthalmic surgery, drug delivery, keratoplasty, vitreoretinal surgery, anti-VEGF, network pharmacology

## 1 Introduction

The fourth volume of *Updates in ocular therapeutics and surgery, volume IV* showcases how our field is advancing along three converging fronts: (i) surgical techniques and visualisation, (ii) biomaterials and drug delivery, and (iii) pharmacotherapy informed by real-world evidence and data-driven discovery. Together, the nine articles in this Research Topic—spanning the cornea, lens and retina and including one surgical methods article—highlight pragmatic solutions to persistent clinical bottlenecks and point to platforms with cross-disease applicability. A Corrigendum in this Research Topic also underscores the value of transparent corrections without altering scientific conclusions.

## 2 Surgery and visualisation

### 2.1 Refining corneal endothelial surgery

Cheong (a) et al. directly compared Descemet membrane endothelial keratoplasty (DMEK) insertion techniques in an Asian cohort—“injector” (endothelium-out) vs. “pull-through” (endothelium-in). Adjusted analyses showed comparable overall clinical outcomes, with surgical indication (not technique) chiefly driving graft failure, and pseudophakic bullous keratopathy faring worse than Fuchs dystrophy. A linked Corrigendum corrected the order of the panels in a figure and did not change the conclusions. These data will help surgeons choose instruments and manoeuvres based on ocular context rather than fashion, and will set expectations for case selection and training.

### 2.2 Stabilising the capsular bag after trauma

Li et al. reported on the long-term outcomes of using heat-shaped polypropylene “capsular hooks” to secure zonular-deficient capsular bags in cases of traumatic crystalline lens subluxation. Visual acuity improved markedly with well-centred IOLs and no device dislodgement over a mean follow-up of ~10-month. In settings without commercial fixation devices, this customisable, low-cost approach is both elegant and scalable.

### 2.3 Retrieving posteriorly dislocated IOLs—simple, safe, reproducible

Gu et al. (Methods article) describe a technique for elevating posteriorly dislocated intraocular lenses by connecting a 22-gauge intravenous catheter to the vitreotome aspiration line, thereby providing controlled suction that lifts the IOL optic away from the retinal surface for safe grasping. In four consecutive cases, the IOL never fell back, and no intraoperative complications occurred. This technique avoids perfluorocarbon liquid (PFCL)-related risks and illustrates how incremental engineering can mitigate risk during delicate vitreoretinal manoeuvres.

### 2.4 A safer way to see: a lutein-based vital dye

Rossi, Gesualdo, Corte et al. piloted the use of a lutein-based vitreous dye in idiopathic epiretinal membrane surgery. The dye selectively stains the vitreous/posterior hyaloid, facilitates key manoeuvres, and is associated with improved visual acuity and reduced central retinal thickness at 4–6 months without IOP penalty. As visualisation increasingly relies on adjuncts with favourable safety profiles, lutein-based dyes may offer a thoughtful alternative to triamcinolone or indocyanine green.

## 3 Biomaterials and delivery

### 3.1 Nanoparticles that can be seen—and deliver

Raîche-Marcoux et al. synthesised fluorescent gold nanoparticles with properties comparable to those of their non-fluorescent counterparts and tracked their localisation across ocular tissues after topical application in *ex vivo* rabbit eyes. The work is a step towards non-invasive delivery systems that can be imaged end-to-end—from the corneal surface to the posterior segment—supporting rational formulation and pharmacokinetic studies.

### 3.2 Harnessing amniotic membrane proteins via smart hydrogels

Basasoro et al. tested two hydrogel platforms loaded with amniotic membrane protein extract in a rabbit model of severe alkali burn. Treated corneas showed more frequent wound closure by day 14 and histologic signals of modulated inflammation. The message is pragmatic: biocompatible, muco-adhesive hydrogels can turn a proven biological milieu (AM proteins) into a controllable, residence-time-enhancing therapy.

## 4 Pharmacotherapy and data-driven discovery

### 4.1 Real-world anti-VEGF: structure–function matters

Zhang C. et al. evaluated aflibercept for diabetic macular oedema with both OCT/OCTA and microperimetry. Short-term treatment reduced central retinal thickness and improved best-corrected visual acuity and fixation stability, while FAZ area/density metrics remained unchanged—reminding us that functional endpoints can move even when angiographic surrogates do not, and supporting the use of microperimetry as a sensitive complement to routine care.

### 4.2 Brolucizumab in practise

Rossi, Gesualdo, Marano et al. presented 1-year, real-world results for brolucizumab in the treatment of neovascular AMD, reporting improved visual acuity and central retinal thickness with relatively few injections and only one intraocular drug-related adverse event. These pragmatic data will help clinicians balance the potency, durability and safety when individualising anti-VEGF regimens.

### 4.3 Data-driven discovery meets traditional medicine

Zhang H. et al. constructed an integrated bioinformatics-network pharmacology-machine-learning framework to investigate three traditional Chinese medicines for diabetic retinopathy. They identified a stigmasterol–PPARG axis and validated this *axis in vivo*. In addition to the specific candidates, their blueprint is noteworthy: AI-assisted target/component prioritisation funnelled into experimental confirmation can accelerate hypothesis generation for complex polypharmacy systems.

## 5 Where do we go from here?

Three priorities emerge. First, we must *standardise* technique-sensitive procedures (e.g., DMEK) alongside indication-aware pathways and training metrics. Second, we need to *translate* biomaterials with imageable, muco-adhesive, and tissue-specific properties from bench to clinic via pharmacokinetics and manufacturability studies. Third, we must *enrich* routine outcomes with function (e.g., microperimetry, fixation stability), not just structure, and continue to develop data-science pipelines that triage candidates before costly trials. The articles assembled here exemplify clinically grounded innovation—small changes that make surgery safer, smarter matrices that keep drugs where they matter, and pragmatic evidence that helps us to treat the right patient with the right tool at the right time.

